# Weight Loss in Cancer and the 2017 Common Terminology Criteria for Adverse Events—Dangerous and Misleading

**DOI:** 10.1002/jcsm.13754

**Published:** 2025-03-06

**Authors:** Aynur Aktas, Kunal C. Kadakia, Jake Waldman, Declan Walsh

**Affiliations:** ^1^ Department of Supportive Oncology, Levine Cancer Institute Atrium Health Charlotte North Carolina USA; ^2^ Department of Medical Oncology, Levine Cancer Institute Atrium Health Charlotte North Carolina USA; ^3^ Hemby Family Endowed Chair in Supportive Oncology, Levine Cancer Institute Atrium Health Charlotte North Carolina USA

**Keywords:** adverse event, cancer, clinical trials, sarcopenia, toxicity, tyrosine kinase inhibitors, weight loss

## Abstract

Accurate toxicity reporting in cancer clinical trials is necessary to promote regulatory decision making. Up to 85% of those with some cancers (e.g., oesophagus, head and neck, and pancreas) experience significant progressive weight loss (WL) throughout treatment. Therefore, it is necessary that investigators accurately grade and report WL. We have identified two dangerous and misleading approaches in the CTCAE specific to WL: (1) severity grades and associated clinical descriptions and (2) lack of pretreatment weight and longitudinal weight changes. This review highlights the critical importance of a more accurate, comprehensive and patient‐focused WL assessment in oncology clinical trials and the CTCAE. We offer a path forward for identification and management of this life‐threatening phenomenon, whether it is due to cancer or an antitumor treatment. The National Cancer Institute should consider the following five ideas and concrete action items for future CTCAE versions: (1) Weight assessment has important implications for toxicity determination and prognosis in clinical trials and should be consistently integrated into study reports; (2) Adverse event assessment should be patient‐focused and supported by clinically relevant definitions of each grade level; (3) the CTCAE grades and associated clinical descriptions for WL should be revised to better reflect the impact of WL on trial endpoints and patient safety; (4) WL should be assessed as a continuous variable to capture duration, severity, and trajectory; and (5) WL should be urgently incorporated into the PRO‐CTCAE to define the individual impact.

## 1 Adverse Event Definition

Adverse events (AEs) are unintended or undesirable experiences (e.g., signs, symptoms and abnormal laboratory tests) that occur in a person exposed to any medical intervention. AEs are monitored in all interventional cancer clinical trials with the 2017 National Cancer Institute's Common Terminology Criteria for Adverse Events version 5 (CTCAE) [1]. The CTCAE are used worldwide. Currently, AEs are graded and reported during trials to delineate potentially tolerable injury or illness from life‐threatening events. The five grades include mild (Grade 1), moderate (Grade 2), severe (Grade 3), life threatening (Grade 4) or death (Grade 5).

Cancer clinical trials evaluate the efficacy, safety and tolerability of established and novel drugs. Drug safety has been examined through clinical and laboratory evaluation in Phase I studies to determine maximum tolerated dose via dose limiting toxicity. Accurate toxicity reporting assists regulatory decision making. Treatment‐induced toxicities can have significant clinical consequences, including hospitalization or treatment modifications, which can impact morbidity, drug efficacy and mortality.

## 2 Current Weight Loss Assessment in Oncology Clinical Trials

In most clinical trials, weight loss (WL) is recorded as an AE when it occurs during or shortly after a specific treatment regimen. This approach aligns with the general definition of an AE as an unintended consequence of medical intervention. However, we argue that this narrow focus on treatment‐associated WL is inadequate and fails to capture the multifactorial nature of WL in the cancer care continuum. This underscores the need for a comprehensive approach to its assessment and management. We have identified two dangerous and misleading issues in the CTCAE specific to WL:

1. Severity grades and associated clinical descriptions
2. Lack of pretreatment weight and longitudinal weight changes

This commentary highlights the critical importance of a more accurate, comprehensive and patient‐focused WL assessment in oncology clinical trials and the CTCAE.

## 3 Multifactorial Nature of WL in Cancer

Involuntary WL is a common symptom associated with cancer and often signifies progressive disease. Contrary to previous assumptions, it can be present even in early‐stage disease or at diagnosis [2]. The prevalence varies by cancer type, with upper gastrointestinal and lung cancers showing higher rates of WL at diagnosis. WL can be both a side effect of treatment and a consequence of cancer itself. This complex interplay presents a challenge in accurately attributing and grading WL as an AE. For example, nausea and vomiting from emetogenic therapies and odynophagia from chemoradiation to the oropharyngeal region or oesophagus can cause reduced caloric intake. Disease‐related factors like malabsorption in pancreatic cancer or metabolic changes induced by the tumour itself can also directly contribute to WL. Notably, up to 85% of those with some cancers (e.g., oesophagus, head and neck, and pancreas) experience significant progressive WL throughout treatment [3]. WL is associated with shorter overall survival independent of body mass index, primary cancer site, disease stage and performance status [4]. Therefore, it is necessary that clinicians and investigators accurately grade and report WL.

This review focuses specifically on WL as an AE in oncology clinical trials. However, it is important to note the interrelated nature of WL, cancer‐associated malnutrition (CAM) and cachexia. WL is often the most visible manifestation of CAM, which can progress to cachexia in advanced stages. For the purposes of AE reporting in oncology clinical trials, we are primarily concerned with accurately capturing and grading WL itself.

Attitudes to WL intervention amongst medical oncologists and haematologists vary [5]. Several factors shape these attitudes: Many clinicians view WL as an unavoidable consequence of cancer and its treatment, leading to a passive approach rather than proactive intervention. There is a prevalent misconception that WL in overweight or obese patients (who make up > 70% of Western populations) is benign or even beneficial. This overlooks the fact that reliance on body mass index (BMI) alone can mask significant changes in body composition, particularly loss of lean body mass in normal or overweight patients.

## 4 Constitutional Symptoms

WL is often grouped with other nonspecific symptoms such as fatigue, anorexia and low‐grade fever under the umbrella term ‘constitutional symptoms’. This vague, secondary status assigned to WL contributes to the inadequate attribution and management of WL. This ambiguity often leads to an underestimation of WL importance, as it may be viewed as a ‘general’ symptom rather than a specific concern. This in turn can also lead to delayed nutritional interventions. To address these issues, we recommend a more nuanced approach to WL as a distinct and important AE, separate from other constitutional symptoms. A shift in perspective is needed to consider WL in conjunction with other functional and quality of life measures. This discussion adds depth to our argument for a more specific approach to WL assessment and reporting in oncology clinical trials and practice.

## 5 WL Assessment and Body Composition

The first concern is that the current CTCAE grading system focuses solely on total body WL and does not account for changes in body composition, which can significantly impact treatment tolerance and prognosis. WL in cancer patients often disproportionately affects lean body mass compared to fat tissue. There is potential increase in fat mass, especially in some cancer types or treatments. These changes can occur even when total body weight remains relatively stable or shows only modest decreases. These highlight the importance of assessing body composition changes beyond just total body weight. The CTCAE is particularly problematic for overweight or obese patients, because significant sarcopenia (skeletal muscle loss) may occur without triggering CTCAE grade thresholds, sarcopenic obesity (low muscle mass with high fat mass) can develop and treatment toxicities and poor outcomes associated with sarcopenia may be overlooked.

The increasing prevalence of overweight and obesity in North America and Europe adds complexity to the assessment of WL in cancer patients. Systemic anticancer therapies can vary significantly in their lipophilicity, which may influence both their efficacy and side effect profiles. Lipophilic drugs may have increased distribution in adipose tissue, potentially affecting their pharmacokinetics and pharmacodynamics in overweight or obese patients. Changes in body composition during treatment could alter drug distribution and effectiveness over time. The relationship between weight, treatment efficacy and toxicity is likely bidirectional. Although WL can impact treatment outcomes, different therapies may also have varying effects on weight and body composition.

A large multinational survey found that a third of respondents would wait for WL of 15%–20% before considering any nutritional intervention [6]. WL of 15%–20% is far from mild or moderate. To put this in perspective for an individual who is 170 cm (5′7″) tall and weighs 70 kg (154 lbs), with a BMI of 24.2. A WL of 10.5–14 kg (23–31 lbs) would result in a new weight range of 56‐59.5 kg (123‐131 lbs). This significant change would lower their BMI to a range of 19.4–20.6, moving them from the upper end of the ‘normal weight’ category to the lower end. Such a substantial WL not only represents a 15%–20% reduction in body weight but also demonstrates a notable shift in body composition, often leading to severe depletion of skeletal muscle mass (sarcopenia). This degree of muscle loss can significantly impact a patient's functional status, quality of life and ability to tolerate cancer treatments. Therefore, clearer guidelines for when to intervene based on WL, rather than waiting for it to become severe, are needed.

## 6 WL Grades

The second CTCAE concern is the approach to WL grade and the associated clinical descriptions (Table 1). The CTCAE grades combine both AE severity and the functional interference experienced by a patient. The CTCAE defines WL grades based on percentage loss from baseline, but these cutoffs significantly underestimate the impact on clinical outcomes. For WL, if a person has lost 5% to < 10% of their weight from trial enrollment but is not on any nutritional intervention, this is a Grade 1 AE. This significant degree of WL is not mild and should, in fact, be alarming, as it is well known to be clinically significant and requires nutritional intervention [7].

**TABLE 1:** Weight loss^a^ in the Common Terminology Criteria for Adverse Events Version 5.0.^b^

Grade	Grade 1	Grade 2	Grade 3	Grade 4
Weight Loss Degree	5% to < 10% from baseline^c^; clinical or diagnostic observations only; Intervention not indicated	10% to < 20% from baseline^c^; nutritional support indicated	≥ 20% from baseline^c^; tube feeding or TPN indicated	Not defined

Abbreviation: TPN, total parenteral nutrition.

^a^ Grade 5 (death) is not appropriate for some AEs and therefore is not an option.

^b^ Adopted from the National Cancer Institute Common Terminology Criteria for Adverse Events Version 5.0.

^c^ At the time of trial enrollment.

When WL reaches 10%–20% and/or nutritional support is indicated, this is still only a Grade 2 AE. This approach is flawed because it conflates the severity of WL with the treatment provided, rather than focusing on the physiological impact of WL itself. Grades 1 and 2 AE are often not reported in toxicity assessment, yet in the case of 10%–20% WL, it is a metabolic crisis. Only when a person has lost ≥ 20% of body weight, is hospitalized, receives tube feeding or is on total parenteral nutrition is WL considered a Grade 3 AE. This approach assumes universal availability of nutrition support services, which is not the case globally. The availability of nutrition support services in cancer centres varies widely worldwide, ranging from nonexistent to comprehensive. This disparity means that patients with identical degrees of WL may be graded differently based solely on their access to nutritional interventions. WL severity may be underreported in settings where advanced nutritional support is not available. Notably, Grade 4 WL is entirely undefined.

These cutoffs between severity grades are naturally arbitrary, but the associated interventions do not align with nutrition management guidelines for cancer patients and are ambiguous [8, 9]. Ambiguity lies in lack of clarity as to whether the associated clinical descriptions are a response to drug toxicity or constitute a criterion for the AE grade. By refocusing the assessment of WL on its actual impact on clinical outcomes, rather than arbitrary percentage thresholds, we can better capture its true significance as an AE. This approach would not only improve the accuracy of toxicity reporting in clinical trials but also guide more timely and appropriate interventions to mitigate negative effects of WL.

## 7 Longitudinal WL

The second CTCAE concern is that the criteria do not consider WL as a continuous variable. For example, pretreatment WL, which is both prevalent and clinically significant [10], is not a component of the WL grading. It has been known (for decades) that up to 80% of individuals experience WL before any cancer diagnosis [11]. Mortality risk depends both on the WL degree and prediagnosis BMI [4]. WL prior to diagnosis of as little as 2.4% is associated with shorter survival (independent of BMI) [4]. The current grading system is specifically ill‐equipped to reflect this ongoing process, as it only offers a snapshot at a single point in time.

WL is often chronic and can transcend the observation period of many clinical trials (both before and after). It is crucial to emphasize that the impact of WL in cancer patients is cumulative, occurring across multiple stages of the disease trajectory. Therefore, a complete longitudinal record of WL is essential to capture this effect. For example, unintentional WL ≥ 5% over the past 6 months can be an early sign of cachexia. As suggested by the 2011 International Cachexia Consensus, it is important to identify cachexia early to integrate nutritional or pharmacological interventions to prevent progression [7]. WL does not spontaneously resolve and is notoriously difficult to improve with any current intervention absent a therapy that alters the natural history of disease. Furthermore, if WL is misattributed as an AE to experimental treatment (rather than the cancer), this could misrepresent the benefit (or harm) of a novel drug [12].

To address these limitations, we suggest the WL grades developed by Martin et al. [4] would be a suitable and valuable amendment to the CTCAE. This method categorizes patients into grades ranging from 0 to 4. At baseline, Grade 0 represented those with the highest BMI and lowest WL percentage, and Grade 4 comprised individuals with the lowest BMI and greatest WL percentage. Grade 0 is associated with longer survival, and Grade 4 is associated with shortest survival. By incorporating both WL percentage and BMI, the grading system accounts for the differential impact of WL based on a person's initial body composition, as highlighted by Fearon et al. [7]. Because these grades are directly linked to mortality outcomes and have been validated in multiple studies, they offer a robust assessment tool that could be standardized across oncology clinical trials.

## 8 WL Reports

There is also bias in reporting AE to regulatory bodies and in publications. ‘Low grade’ AE (Grades 1 and 2) constitute over 50% of toxicity, and their impact is often detrimental [12]. Such misclassification lends to minimizing language including ‘acceptable’, ‘manageable’ and ‘tolerable’ when describing severe AE [13]. Such terms are imprecise and, when applied specifically to WL, are generally misleading and clinically dangerous. It is well established that WL ≥ 15% (currently Grade 2) impairs physiological function, quality of life and overall cancer survival [14]. However, these toxicities are usually not reported in clinical trial reports, as they are not required for FDA drug approval.

WL is often ignored in oncology trials and frequently evaluated with a nonspecific classification. We reported an analysis of 153 consecutive publications in three major oncology US journals from 2022 related to systemic antitumor treatments [15]. Ninety percent failed to report any nutrition status data such as presence of malnutrition and pretreatment weight. When reported, data were limited to either BMI at trial enrollment or pretreatment weight changes (gain or loss) [15]. Importantly, systematic screening for CAM risk was absent in all 153. WL is therefore an overlooked AE in both toxicity assessment and pivotal trial endpoint reports. Weight data provide important and distinct prognostic information and should be routinely reported.

## 9 Systemic Anticancer Therapies

In the era of novel targeted therapies, more cancer treatments are given continuously [16]. The CTCAE also fails to reflect longitudinal WL trajectory and impact throughout the illness that might secondarily influence drug pharmacokinetic profiles and patient reported outcomes (PRO). For example, tyrosine kinase inhibitors (TKI) often requires prolonged treatment duration and have chronic significant side effects outside of the initial study window [17]. The TKI pathways involve inhibition of mitogen‐activated protein kinase and extracellular signal‐regulated kinases, which prevent induction of muscle anabolism [18]. Because major toxicities from this muscle‐eroding mechanism can cause sarcopenia, this could also remain undetected—particularly in overweight or obese patients—under the current CTCAE.

Although TKI can improve cancer survival, they are known to paradoxically contribute to WL. For example, in the CORRECT Trial [19], the prevalence of WL (any grade) in individuals randomized to receive regorafenib was 14%, but only 2% in the placebo arm. This drug was approved based on a 1.4‐month median overall survival advantage. This was a statistically significant but arguably clinically marginal difference. The known prognostic impact of CAM on cancer prognosis and outcomes was not considered.

Recent literature has shed light on the myotoxicity associated with cancer treatments, leading to significant muscle loss during certain regimens [20]. This phenomenon extends beyond TKI and affects a broad range of cancer therapies. Different treatment regimens can have distinct impacts on muscle and fat tissue. For example, in pancreatic cancer patients, FOLFIRINOX is strongly associated with muscle loss, and Gemcitabine‐abraxane is associated with fat loss [21].

## 10 Patient‐Reported Outcomes

WL is also a major source of distress for cancer patients and their caregivers, as it is an obvious manifestation of disease and often signifies progressive disease and the proximity of death. Real‐time integration of PRO advances engagement with clinical trials, optimizes toxicity assessment and enhances outcomes like quality of life [22]. In response to underestimation of AE or lack of engagement by clinician‐investigators, the National Cancer Institute (NCI) created the PRO‐CTCAE item library [23]. This system allows patients to describe their symptoms for frequency, severity and interference with daily activities. The PRO‐CTCAE, while including symptoms that may indirectly impact nutritional status, does not explicitly link these to WL. It is noteworthy that WL is entirely absent from the item library even though it is both a symptom (subjective) and a sign (objective) of disease. Given WL prevalence, severity and biological importance in cancer patients, this is an extraordinary omission.

## 11 Recommendations

The NCI should consider the following five ideas and concrete action items for future CTCAE versions and how AE are reported (especially as cancer treatment shifts away from cytotoxic therapies).

1. Weight assessment has important implications for toxicity determination and prognosis in clinical trials and should be consistently integrated into study reports [24].
  - Create a cumulative WL tracking system that considers pretreatment weight and weight changes throughout clinical trials
  - Develop standardized protocols for computed tomography‐based muscle and fat measurements
  - Revise publication guidelines to mandate reporting of all severity grades of WL
2. AE assessment should be patient‐focused and supported by clinically relevant definitions of each grade level.
  - Partner with patient advocacy groups to design PRO tools that capture functional status, fatigue and quality of life changes related to WL.
3. The CTCAE grades and associated clinical descriptions for WL should be revised to better reflect the impact of WL on trial endpoints and patient safety.
  - Incorporate the Martin et al. [4] WL grading system, which accounts for both BMI and percentage WL, into the CTCAE.
  - Lower the threshold for WL to be considered an AE
4. WL should be assessed as a continuous variable to capture duration, severity and trajectory. Efforts should be made to monitor WL as a distinct AE.
  - Develop tools to track WL duration, severity and trajectory
  - Establish protocols for monitoring WL as a distinct AE
  - Train clinical trial staff on proper WL monitoring and documentation
5. WL should be urgently incorporated into the PRO‐CTCAE to define the individual impact.
  - Develop BMI‐adjusted WL assessment tools for the PRO‐CTCAE
  - Conduct pilot studies to assess the effectiveness of the new PRO‐CTCAE WL module

We hope this commentary will shed light on the current significant shortcomings of the CTCAE in relation to WL. The forthcoming iteration of these criteria should incorporate our recommendations and undergo appropriate adjustments. We offer a path forward for identification and management of this life‐threatening phenomenon, whether it is due to cancer or an antitumor treatment.

## Conflicts of Interest

The authors declare no conflicts of interest.
